# A scoping review of outcome reporting in randomized controlled trials of incisional and non-incisional ventral hernia repair

**DOI:** 10.1007/s10029-025-03553-y

**Published:** 2026-01-16

**Authors:** Haiye Shen, Dominic Farris, David L. Sanders, Helen Dawes, Sarah E. Lamb, John M. Findlay

**Affiliations:** 1 Department of Clinical and Biomedical Sciences, Faculty of Health and Life Sciences, University of Exeter, Exeter, EX1 2HZ UK; 2 NIHR Exeter Biomedical Research Centre, St Luke’s Campus, University of Exeter Medical School, Exeter, EX1 2HZ UK; 3 Public Health and Sport Sciences, Faculty of Health and Life Sciences, University of Exeter Medical School, Exeter, EX1 2HZ UK; 4 Academic Department of Abdominal Wall Surgery, North Devon District Hospital, Royal Devon University Healthcare NHS Foundation Trust, Barnstaple, EX31 4JB UK

**Keywords:** Incisional hernia, Hernia, Ventral hernia, Outcome reporting, Hernia repair, Randomized controlled trials

## Abstract

**Background:**

Incisional and non-incisional ventral hernias are common and important causes of symptoms, functional restriction, and complications, with significant potential to impact upon quality of life. The goals of hernia repair are to treat, prevent or improve these. However, outcomes of surgery remain relatively poor with significant gaps within the evidence base, which may be due to inconsistent use of outcome measures. The aim of this study was to appraise outcome reporting in the recent literature of randomized controlled trials (RCTs).

**Objectives:**

This scoping review aimed to map and categorize the outcome measures reported in RCTs of incisional and non-incisional ventral hernia repair.

**Eligibility criteria:**

All RCTs assessing any intervention related to incisional, primary, or recurrent ventral hernia repair between2015 and 2025 were included.

**Source of evidence:**

A literature search was performed of the PubMed, EMBASE (1974 to present), and Cochrane Central Register of Controlled Trials databases in November March 2025.

**Charting methods:**

Data was extracted independently by two reviewers. All outcomes reported by the included studies were identified and recorded.

**Results:**

118 RCTs were included. Their outcomes were mapped into five main broad categories. The commonest outcomes used were short-term operative complications (72.9%), hernia recurrence (59.3%), pain (57.6%), and quality of life (33.9%). Patient-reported outcomes were measured in 78 (66.1%) randomized controlled trials, of which 15 assessment tools were identified; 11 were generic, and 4were hernia-specific. There was considerable heterogeneity in how and when these endpoints were assessed and defined.

**Conclusion:**

This scoping review found considerable differences in outcome reporting in contemporary RCTs of incisional and non-incisional ventral hernia. These have significant implications for translating evidence into practice, and its synthesis, and support the need for a core outcome set in this field. However, we identified areas such as abdominal wall function which are infrequently reported and require consideration.

**Supplementary Information:**

The online version contains supplementary material available at 10.1007/s10029-025-03553-y.

## Introduction

 Incisional and primary (non-incisional) ventral hernias are common conditions that significantly impact patient symptoms, complications, and overall quality of life (QoL). Primary ventral hernias—most commonly umbilical and epigastric—affect up to 25% of patients [1], and are frequently treated surgically [2]. Abdominal incisional hernias, meanwhile, occur in approximately 12% of patients within two years of abdominal surgery, with more than two-thirds eventually requiring surgical repair [3].

Despite the high prevalence and clinical importance of these hernias, surgical outcomes remain highly variable. Recurrence rates for both primary and incisional hernias consistently exceed 12% [4, 5]. However, while recurrence is often the default outcome measured in hernia research, it represents only one aspect of post-operative recovery. Outcomes are shaped by a range of post-surgical factors, many of which remain inconsistently reported.

A recent systematic review of studies on incisional hernia repair published between 2010 and 2019 revealed substantial variation in both the outcomes assessed and the methods used to report them [6]. This highlights a lack of standardization in outcome reporting across studies. Therefore, this study aims to provide a current and comprehensive evaluation of outcome reporting practices in randomized controlled trials (RCTs) of both incisional and primary ventral hernia repairs, and to map the reported outcome measures to key aspects of clinical trial outcomes.

## Methods

This scoping review was conducted on the basis of a pre-established protocol, which was developed in accordance with the Preferred Reporting Items for Systematic Reviews and Meta-Analyses (PRISMA) guidelines [7], and was initially registered on PROSPERO (Registration ID: CRD420251006980). It was subsequently conducted as a scoping review based on its objective and methodology.

### Eligibility criteria

All RCTs (published or protocol) assessing any intervention before, during, or after incisional, primary, or recurrent ventral hernia repair between 2015 and 2025 were included. Studies of groin hernias, hiatal hernias, and parastomal hernias, as well as studies designed to prevent incisional hernias, were excluded.

### Search strategy

PubMed, EMBASE (1974 to present), and Cochrane Central Register of Controlled Trials (CENTRAL) databases were searched using the following four groups of search terms: (incisional OR ventral OR epigastric OR umbilical OR port OR flank OR lumbar), (hernia OR abdominal wall), (reconstruction OR repair), (randomized controlled trial OR controlled clinical trial) in March 2025, and then updated in November 2025. They were combined with Boolean Operators ‘AND’ to narrow the search results. The search results were limited to human studies published between 2015 and 2025. Bibliographies of retrieved articles will be searched, along with Clinical Trials.gov to identify any further potential studies. All citations were managed using EndNote 21(Clarivate Analytics). Duplicate records were identified and removed using Rayyan, a web-based tool for review management [8].

### Selection of studies

Studies were screened on the basis of title and abstract, and reviewed independently by two reviewers (HS and JMF). The full texts of relevant abstracts were retrieved. Any differences were resolved by discussion.

### Data extraction

Data was extracted independently by two reviewers (HS and JMF). All outcomes reported by the included studies were identified and recorded. In addition to the above endpoints, data were collected regarding study design, population, intervention, and control.

### Study quality

The quality of the included RCTs was evaluated using the Jadad scale, which assesses randomization (2 points), blinding (2 points), and withdrawals/dropouts (1 point) [9]. Higher scores (3, 4, and 5) indicate better methodological quality.

### Data analysis

Outcomes from all studies were extracted, and the number and frequency of each outcome were reported and calculated. Descriptive data, such as study characteristics, were recorded and summarized. All data were entered into Microsoft Excel for management and analysis. Thematic analysis was performed on reported outcomes after coding the themes based on their alignment with key domains of clinical trial outcomes.

## Results

### Literature search

**Fig. 1:**
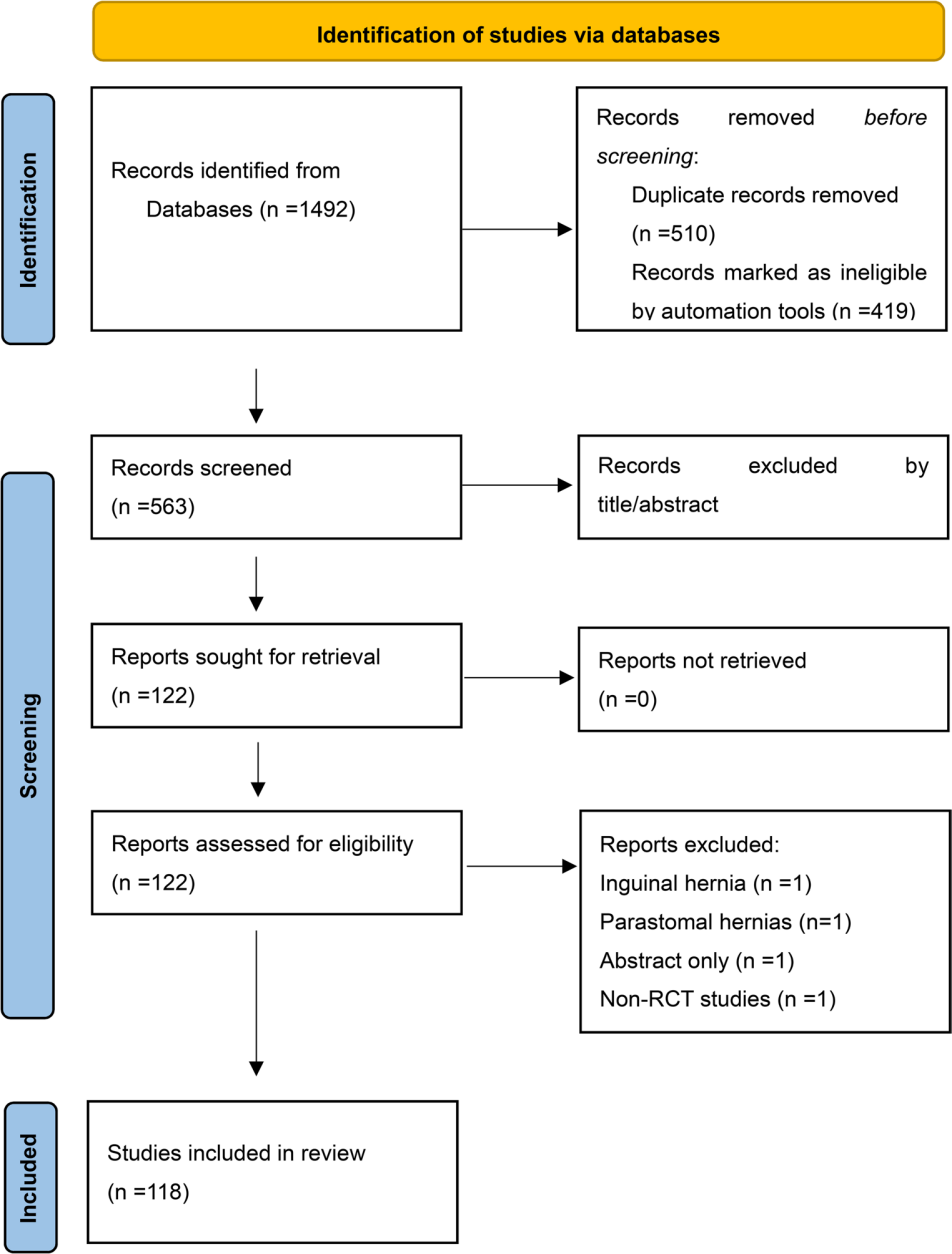
PRISMA diagram of search strategy

A total of 1492 manuscripts were identified in the initial search. After removing duplicate references, and applying the filter function in Rayyan to mark records containing the following exclusion keywords: systematic review, meta-analysis, survey, case report, observational, and non-randomized, 563 studies were screened by title and abstract, of which 122 full texts were retrieved, with 118 studies finally included [10–127] (Fig. 1).

### Characteristics of included studies

These 118 RCTs enrolled a total of 11,863 patients (mean = 104.9, median = 87). Eighty-one (68.6%) were conducted in a single center, and thirty-seven (31.4%) were multicenter. Fifty-two (44.1%) were open-label, thirty-one (26.3%) were single-blind (patients), and thirty-five (29.7%) were double-blind (patients and assessors). The methodological quality of the included RCTs was assessed using the Jadad scale, as shown in Table 1, with scores ranging from 1 to 5 (mean = 3.1, median = 3).

**Table 1:** Study quality

Jaded score	Number of studies (*n* = 118)	Reference
1	7 (5.9)	[45, 70, 80, 102, 105, 106, 119]
2	33 (28)	[10, 13, 16, 23, 24, 35, 44, 58, 60, 65, 71–73, 77, 80, 82, 84, 85, 89, 93, 94, 99, 104, 109, 115–118, 121, 122, 124, 126, 127]
3	32 (27.1)	[11, 12, 14, 15, 17, 25, 28, 32, 36, 38, 43, 47–49, 51, 52, 56, 57, 62, 63, 74, 81, 83, 87, 88, 90, 97, 103, 107, 112, 113, 125]
4	38 (32.2)	[18–22, 26, 27, 29–31, 33, 34, 37, 39–42, 46, 50, 53–55, 61, 64, 66, 68, 69, 76, 78, 91, 92, 95, 98, 101, 108, 111, 114, 123]
5	8 (6.8)	[59, 67, 75, 86, 96, 100, 110, 120]

### Population

These 11,863 adult patients underwent repair of incisional hernia (35.5%), umbilical (11.1%), epigastric (2.7%), and mixed (50.7%). Their ages ranged from 18 to 86 years, with overall sex distribution being 52.3% male and 47.7% female.

### Intervention and controls

Interventions were categorized into the following domains: surgical approach in 49 RCTs (41.5%), mesh type in 16 (13.6%), mesh fixation method in 20 (16.9%), postoperative management in 14 (11.9%), anesthesia/analgesia methods in 15 (12.7%), and preoperative preparation in 4 (3.4%).

Controls were either standard care in 11 (9.3%), or a specified intervention in 107 (90.7%). The methodological characteristics of the included studies were summarized in Supplementary Table 1.

### Outcomes

**Fig. 2:**
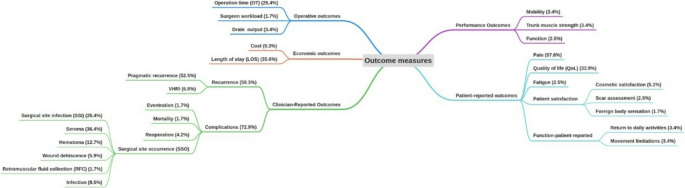
Categorized outcome measurements reported in the returned literature

The outcomes reported varied, and included pain, recurrence, complications, length of stay (LOS), operation time (OT), quality of life (QoL), drain output, trunk muscle strength, function, patient satisfaction, cosmetic satisfaction, scar assessment, movement limitation, mobility, return to daily activities, fatigue, cost, and surgeon workload. These outcomes were mapped into five main broad categories based on their relevance to key aspects of clinical trial outcomes, which are shown in Fig. 2. In addition, we also performed an exploratory mapping to the Outcome Measures in Rheumatology (OMERACT) framework [127]. This framework includes four core areas: life impact, resource use, pathophysiological manifestations, and death. Many of the outcomes identified in our review showed partial alignment with these domains. For example, life impact (pain, QoL, patient satisfaction), resource use (LOS, cost, and surgeon workload), and pathophysiological manifestations (recurrence, complications). This framework was not used to guide our primary analysis, but is presented here to illustrate potential compatibility with established frameworks.

### Operative outcomes

Thirty (25.4%) studies reported on operating time, two (1.7%) on surgeon workload, and four (3.4%) on surgical site drain output. The operating time was recorded during surgery. Surgeon workload was assessed using the NASA-TLX scale, and the modified SURG-TLX scale. Drain output was assessed at daily and total amounts.

### Economic outcomes

Forty-two (35.6%) studies reported on LOS, and eleven (9.3%) reported on hospital resource use (i.e., total cost).

### Clinician-reported outcomes

Seventy (59.3%) studies reported on recurrence, and eighty-six (72.9%) on complications. Recurrence was assessed radiologically (using CT) in six (5.1%) studies, clinically in thirty-five (29.7%) studies, by ultrasound in two (1.7%) studies, by Ventral Hernia Recurrence Inventory (VHRI) in eight (6.8%), and mixed method (combination of any two or three of CT, clinical examination, and ultrasound) in nineteen (16.1%) studies (Table 2). There was no consistency in the timepoints of assessment for recurrence, ranging from one week to two years, with long-term follow-up extending to five to ten years. The commonest time point was 12 months postoperatively (*n* = 13, 11%).

**Table 2:** Recurrence assessment

Variable	Number of studies (*n* = 118)	Reference
Radiological (CT)	6 (5.1)	[26, 27, 46, 49, 57, 89]
Clinical examination	35 (29.7)	[12, 15, 18, 23, 25, 32, 35, 38, 41, 43, 47, 48, 54, 56, 58, 60, 61, 65, 72, 78–80, 82, 84–88, 90, 99, 101, 106, 107, 119, 124]
Ultrasound	2 (1.7)	[17, 34]
VHRI	8 (6.8)	[10, 21, 33, 42, 44, 57, 73, 91]
Mixed method	19 (16.1)	[31, 37, 39, 59, 63, 64, 92, 93, 96–98, 108–110, 116–118, 120, 126]

Complications in the included RCTs were mapped into four categories: surgical site occurrence (SSO), reoperation, eventration, and mortality. According to the ventral hernia working group classification, surgical site infection (SSI), seroma, wound dehiscence, retromuscular fluid collection (RFC), and infection were mapped under SSO. Although complications were frequently reported as outcome measures, pre-defined definitions were the exception. Five (4.2%) studies declared pre-definitions of SSI, five (4.2%) for seroma, one (0.8%) for hematoma, one (0.8%) for wound dehiscence, one (0.8%) for RFC, one (0.8%) for reoperation, and one (0.8%) for eventration (Table 3). These complications were evaluated either clinically or with specific assessments (Table 4), from one week to two years, where six months and one year were the most frequently used timepoints.

**Table 3:** Definitions of complications

Complications	Definitions	Reference
SSI	Diagnosed as defined by Centre for Disease Control	[30, 71]
	Classified according to the Centre for Disease Control, as superficial, deep, and organ space infection	[28]
	Defined, per the U.S. Centers for Disease Control and Prevention guideline	[26]
	Defined according to the Ventral Hernia Working Group (VHWG) definitions, including superficial and deep incisional SSI	[101]
Seroma	Collection of serosanguineous fluid more than 50 ml in subcutaneous plane after surgery	[35]
	Delimited fluid collection located in the abdominal wall with dimensions > 1 cm in the transverse, coronal, and sagittal planes	[50]
	Even small fluid formations on the 14th POD (1 cm³) were considered to be a seroma formation	[25]
	Defined as a fluid collection detected by palpation on clinical examination	[92]
	Defined as a fluid collection in relation to the previous hernia sac or in relation to the mesh	[111]
Hematoma	Defined a hematoma as a solid swelling of clotted blood within the tissues	[27]
Wound dehiscence	Defined as an opening of the surgical incision of any size	[27]
RFC	Volumes of fluid collections, hematoma or seroma in the retromuscular space, behind the linea alba and anterior rectus sheath	[19]
Reoperation	Unanticipated return to the operating room for complications related to the index operation	[26]
Eventration	Standard laparoscopic repair with bridging of the fascial defect is sometimes associated with bulging or clinical eventration	[47]

**Table 4:** Complications assessments

Complications	Number of studies (*n* = 118)	Assessments
SSI	30 (25.4)	Southampton score, CDC classification, clinical examination
Seroma	43 (36.4)	Clinical examination, ultrasound
Hematoma	15 (12.7)	Clinical examination, ultrasound
Wound dehiscence	7 (5.9)	Clinical examination
RFC	2 (1.7)	Ultrasound
Reoperation	5 (4.2)	Medical record
Infection	10 (8.5)	CDC classification, clinical examination
Eventration	2 (1.7)	CT
Mortality	2 (1.7)	Medical record

### Performance outcomes

Six (5.1%) studies reported on mobility, function, and trunk muscle strength. The outcomes measured were objective assessments. Mobility was evaluated using a standardized Time Up and Go (TUG) test at the time points of thirty days, six months, and one year. Function was evaluated within the same timeframe as mobility, but through different assessments, including the five times sit-to-stand (5xSTS) test and the quantitative, continuous abdominal core function assessment (QUeST). Trunk muscle strength was assessed with an isokinetic dynamometer, and the double leg lowering test.

### Patient-reported outcomes

Seventy-eight (66.1%) RCTs reported on patient-reported outcomes (PROMS), either as a primary or secondary outcome. Patient-reported outcomes identified include pain (*n* = 68, 57.6%), fatigue (*n* = 3, 2.5%), QoL (*n* = 40, 33.9%), patient-reported function (*n* = 4, 3.4%), and patient satisfaction (*n* = 6, 5.1%). A variety of assessment tools were reported in the studies, eleven were general and four were hernia-specific. There was a lack of temporal consistency in these assessments, ranging from thirty minutes to thirty-six months postoperatively.

### Pain

Thirty-nine (33.1%) studies reported pain as a primary outcome and forty-one (34.7%) studies reported pain as a secondary outcome, respectively. A variety of assessment tools were used, including Numeric Rating Scale-11 (NRS-11), Visual Analog Scale (VAS), Verbal Descriptor Scale (VDS), Patient-Reported Outcomes Measurement Information System Pain Intensity Short Form 3a (PROMIS 3a), and Ventral Hernia Pain Questionnaire (VHPQ) (Table 5). Pain was assessed over a wide range of time periods, from six hours to thirty-six months postoperatively, with the most common time point being six months postoperatively.

**Table 5:** Pain assessment tools

Variable	Number of studies (*n* = 118)	Reference
NRS-11	12 (10.2)	[22, 29, 39, 40, 67, 69, 76, 86, 97, 110, 112, 120]
VAS	48 (40.7)	[10, 12–17, 19, 20, 24–27, 32, 34, 36, 38, 41, 44–46, 50, 54, 58, 59, 61, 62, 68, 75, 79, 81, 82, 88, 90, 91, 96, 98–107, 109, 111]
PROMIS 3a	5 (4.2)	[21, 22, 33, 73, 87]
VDS	1 (0.8)	[18]
VHPQ	2 (1.7)	[37, 57]

### Quality of life

Forty (33.9%) studies reported QoL from thirty days to one year postoperatively, with thirty days being the most common assessment time point. A variety of assessment tools were used, including the Hernia-Related Quality-of-Life Survey (HERQLes), European Registry for Abdominal Wall Hernias Quality of Life Score (EuraHS), Carolina Comfort Scale (CCS), Short Form-36 (SF-36), Activities Assessment Scale (AAS), EuroQol 5-Dimension Questionnaire (EQ-5D), and WHO Quality of Life-BREF (WHO QOL-BREF) (Table 6), where HERQLes, EuraHS, and CCS are hernia-specific assessment tools. They all assess pain and movement restriction, where CCS addresses mesh sensation, and EuraHS includes cosmetic concerns. The HERQLes is more comprehensive and assesses psychological Impact, social function, symptom burden, and QoL perception.

**Table 6:** QoL assessment tools

Variable	Number of studies (*n* = 118)	Reference
HERQLes	12 (10.2)	[10, 21, 33, 39, 41, 42, 44, 55, 73, 86, 87, 107]
SF-36	7 (5.9)	[16, 34, 37, 54, 59, 97, 122]
AAS	3 (2.5)	[26, 27, 41]
EuraHS	4 (3.4)	[30, 49, 51, 115]
CCS	10 (8.5)	[11, 20, 54, 58, 82, 85, 89, 98, 110, 111]
WHO QOL-BREF	2 (1.7)	[36, 46]
EQ-5D	2 (1.7)	[37, 76]

### Function – patient-reported

Only four (3.4%) studies addressed patient-reported function, where they measured function in terms of return to daily activities and movement limitation. Return to daily activities was assessed by the six-point daily activity questionnaire, where movement limitation was assessed using VAS.

### Patient satisfaction

Six (5.1%) studies reported on patient satisfaction, classified as cosmetic satisfaction, scar assessment, and foreign body sensation. Cosmetic satisfaction was assessed by patient feedback, where the assessment time point was not specifically stated. Scar Assessment was measured by Patient and Observer Scar Assessment Scale, version 2 (POSAS v2) at thirty days postoperatively. Foreign body sensation was assessed by patient feedback at twelve months postoperatively.

## Discussion

This scoping review aimed to identify all randomized controlled trials (RCTs) conducted over the past 10 years involving both incisional and non-incisional ventral hernias, with a focus on the outcome measures assessed and the methods used for assessment. We identified 118 RCTs, most of which included a mixed population of hernias and investigated either surgical approach or specific operative details (such as mesh type or fixation technique).

The most commonly reported primary outcomes were hernia recurrence, operative complications, and pain. Secondary outcomes frequently included healthcare/resource utilization, pain, QoL, and complications. These outcomes could broadly be categorized as either patient-reported or non-patient-reported, with the latter assessed by surgeons or other clinical evaluators—being more prevalent.

We observed substantial heterogeneity not only in which outcomes were reported, but also in how they were measured, including the assessment techniques and the timepoints at which evaluations were performed. For example, hernia recurrence—a classic surgeon-reported outcome—was inconsistently assessed, ranging from a few weeks to several years postoperatively, and using a variety of methods including clinical examination, ultrasound, cross-sectional imaging (CT), and VHRI. Similarly, operative complications were variably defined and inconsistently reported across studies.

Patient-reported outcome measures (PROMs) were used less consistently, with notable variation in the tools applied. Both generic and hernia-specific PROMs were employed, though no single instrument emerged as dominant. Importantly, we identified gaps in outcome assessment, particularly around the quantitative evaluation of abdominal wall function. While physical function was acknowledged as an important QoL component, it was inconsistently measured, often relying on tests such as the timed up-and-go, sit-to-stand, or isokinetic muscle strength assessments [42, 51, 96]. This represents a key area for future research—specifically, the development of standardized and clinically meaningful tools to evaluate abdominal wall function.

These findings align with previous reviews from 2010 to 2019, which focused solely on incisional hernias and included observational studies [6]. Similar trends have been noted in studies of other visceral [128] and non-abdominal surgeries [129]. Our results reinforce the urgent need to establish and adopt core outcome sets—not only for incisional hernias (e.g. the HarMoNy project currently underway) [130], but also for non-incisional ventral hernias. Given the similar clinical impact of both hernia types, and the consistently suboptimal and heterogeneous reporting of outcomes, standardized outcome measures are essential. Without this, the generalizability of evidence, its synthesis, application in clinical practice, and the ability to design and interpret future trials remain limited [131, 132].

Additionally, we noted that functional outcomes—especially those relating to the abdominal wall—remain relatively underexplored. While some PROMs assess physical function as part of QoL, there is little consistency in how this is done, and very limited use of quantitative assessments. As functional recovery is a major determinant of patient satisfaction and surgical success, developing robust, reproducible tools to measure abdominal wall function should be a priority for future studies.

This review has some limitations. By restricting the search to the past 10 years to ensure contemporary relevance, we may have excluded important earlier studies, limiting our ability to assess longitudinal trends. Furthermore, we used the Jadad score for study quality assessment, rather than more comprehensive tools like the Cochrane RoB 2 tool [133]. However, we believe these limitations do not significantly affect our overall conclusions.

## Conclusion

This scoping review highlights significant variability in the outcomes reported and how they are measured in RCTs of ventral hernia repair. Non–patient-reported outcomes continue to dominate, and the inconsistency in tools and timing of assessment hinders the comparability and applicability of findings. Although hernia-specific PROMs exist, no standard tool has been universally adopted, and critical aspects of recovery—particularly function—remain underassessed. To improve the quality and relevance of future research, there is a clear need to develop and implement standardized, validated outcome measures that reflect the full spectrum of the patient experience.

## Supplementary Information

Below is the link to the electronic supplementary material.

Supplementary Material 1 (DOCX 666 KB)
