# Supplementary material for: A scoping review of outcome reporting in randomized controlled trials of incisional and non-incisional ventral hernia repair
Source: Hernia. 2026 Jan 16;30(1):71. doi: 10.1007/s10029-025-03553-y (PMC12811304; doi:10.1007/s10029-025-03553-y)
Supplement: Supplementary file 1 — Supplementary Material 1 (DOCX 666 KB) [file 10029_2025_3553_MOESM1_ESM.docx]

| **Supplementary Table 1 Methodological Characteristics** | | |
| --- | --- | --- |
| **Variable** | **Number of studies**  **(n=118)** | **Reference** |
| **Centre** |  |  |
| Single | 81 (68.6) | [10, 12-17, 19, 20, 23, 24, 27-29, 31, 32, 35, 36, 38-46, 48, 50-52, 54, 58, 60, 62-64, 67-71, 73-76, 80-86, 88, 92-95, 99-109, 111-115, 121-127] |
| Multicentre | 37 (31.4) | [11, 18, 21, 22, 25, 26, 30, 33, 34, 37, 47, 49, 53, 55-57, 59, 61, 65, 66, 72, 77-79, 87, 89-91, 96-98, 110, 116-120] |
| **Blinding** |  |  |
| Open-label | 52 (44.1) | [10-17, 23-25, 28, 32, 35, 36, 38, 44, 45, 48, 49, 51, 52, 58, 62, 65, 70-73, 79, 81-85, 90, 93, 94, 97, 102, 104-106, 109, 112, 113, 116, 118, 119, 121, 122, 127] |
| Single-blind | 31 (26.3) | [18, 19, 21, 27, 33, 41, 43, 47, 53, 55, 60, 61, 68, 74, 77, 80, 86-89, 91, 92, 95, 96, 99, 108, 111, 115, 124-126] |
| Double-blind | 35 (29.7) | [20, 22, 26, 29-31, 34, 37, 39, 40, 42, 46, 50, 54, 56, 57, 59, 63, 64, 66, 67, 69, 75, 76, 78, 98, 100, 101, 103, 107, 110, 114, 117, 120, 123] |
| **Year of publication** |  |  |
| 2015 | 7 (5.9) | [52, 71, 80, 100, 109, 111, 126] |
| 2016 | 8 (6.8) | [16, 74, 88, 91-93, 119, 121] |
| 2017 | 4 (3.4) | [43, 62, 63, 75] |
| 2018 | 14 (11.9) | [12, 13, 18, 31, 54, 59, 61, 77, 81, 82, 98, 107, 118, 123] |
| 2019 | 3 (2.5) | [96, 117, 125] |
| 2020 | 10 (8.5) | [26, 35, 45, 47, 58, 67, 106, 110, 114, 116] |
| 2021 | 13 (11) | [27, 33, 49, 56, 60, 68, 70, 73, 85, 86, 90, 105, 127] |
| 2022 | 17 (14.4) | [17, 21, 28, 36, 37, 48, 51, 55, 64, 66, 72, 89, 99, 103, 115, 122] |
| 2023 | 19 (16.1) | [11, 14, 15, 22, 24, 25, 32, 34, 39, 40, 42, 50, 65, 69, 79, 87, 101, 108, 112] |
| 2024 | 18 (15.3) | [10, 19, 20, 23, 29, 30, 38, 41, 53, 57, 76, 78, 83, 84, 94, 97, 104, 124] |
| 2025 | 5 (4.2) | [44, 46, 95, 113, 120] |
| **Intervention categorization** |  |  |
| Surgical approach | 49 (41.5) | [10, 11, 13, 16-18, 21, 22, 24-26, 32, 35-38, 43, 44, 47, 49, 50, 53, 56, 57, 59, 61, 68, 72-74, 78, 79, 83, 86, 87, 89, 91, 92, 95, 97, 106, 108, 110, 112, 117, 119-122] |
| Mesh type | 16 (13.6) | [15, 23, 27, 33, 34, 41, 45, 55, 60, 63, 64, 80, 84, 88, 93, 118] |
| Fixation method | 20 (16.9) | [14, 20, 39, 46, 54, 58, 81, 82, 85, 96, 98, 99, 101, 105, 107, 109, 115, 124, 126, 127] |
| Postoperative management | 14 (11.9) | [12, 19, 28, 30, 42, 51, 52, 65, 66, 70, 90, 94, 111, 116] |
| Anesthesia/analgesia methods | 15 (12.7) | [29, 40, 62, 69, 75-77, 100, 102-104, 113, 114, 123, 125] |
| Preoperative preparation | 4 (3.4) | [31, 48, 67, 71] |
| Numbers in parentheses are percentages | |  |
